# The diversity of *Eimeria* spp. in cattle in the Brazilian Semiarid region

**DOI:** 10.1590/S1984-29612022037

**Published:** 2022-07-11

**Authors:** Lídio Ricardo Bezerra Melo, Luana Carneiro Sousa, Brendo Andrade Lima, Ana Luzia Peixoto Silva, Estefany Ferreira Lima, Larissa Claudino Ferreira, Thais Ferreira Feitosa, Vinícius Longo Ribeiro Vilela

**Affiliations:** 1 Programa de Pós-graduação em Ciência e Saúde Animal, Universidade Federal de Campina Grande – UFCG, Patos, PB, Brasil; 2 Departamento de Medicina Veterinária, Instituto Federal de Educação, Ciência e Tecnologia da Paraíba – IFPB, Sousa, PB, Brasil

**Keywords:** Coccidia, gastrointestinal protozoa, mixed production, ruminants, Coccídios, protozoários gastrintestinais, produção mista, ruminantes

## Abstract

The aim of the present study was to find out the diversity of *Eimeria* species in cattle herds in the semiarid region of Brazil. Forty cattle fecal samples were collected from 20 farms in the Paraíba State, Northeast Brazil, and examined by centrifugation-floatation technique in sucrose solution. From each positive animal, 20 oocysts were photographed and measured. Infection by *Eimeria* spp. was detected in 17.12% (137/800) of the samples analyzed. All the farms had at least one animal that was positive for *Eimeria* spp. (100%; 20/20). In total, 2740 coccidia were photographed and measured. The species detected were: *Eimeria bovis* (35.1%); *Eimeria canadensis* (17.48%); *Eimeria auburnensis* (14.7%); *Eimeria ellipsoidalis* (9.7%); *Eimeria zuernii* (7.22%); *Eimeria brasiliensis* (4.56%); *Eimeria bukidnonensis* (3.97%); *Eimeria illinoisensis* (2.91%); *Eimeria wyomingensis* (1.42%); *Eimeria alabamensis* (1.27%); *Eimeria cylindrica* (0.76%); *Eimeria pellita* (0.54%); *Eimeria ildefonsoi* (0.21%); and *Eimeria subspherica* (0.07%). It was concluded that cattle in the semiarid region of Brazil were parasitized by 14 species of *Eimeria*. It is thinked that the sanitary management employed, as well as the system used for raising these animals, is the crucial point that leads to high rates of infection in this region.

## Resumo

Objetivou-se descrever a diversidade de *Eimeria* em rebanhos bovinos do Semiárido do Brasil. Amostras fecais de 40 bovinos foram coletadas, provenientes de 20 fazendas no Estado da Paraíba, Nordeste do Brasil, e examinadas pela técnica de centrífugo-flutuação em solução de sacarose. De cada animal positivo, foram fotografados e mensurados 20 oocistos. A presença de *Eimeria* spp. foi detectada em 17,12% (137/800) das amostras analisadas. Todas as propriedades tiveram ao menos um animal positivo para *Eimeria* spp. (100%; 20/20). No total, foram fotografados e mensurados 2.740 coccídios, cujas ocorrências foram: *Eimeria bovis* (35,1%), seguida de *Eimeria canadensis* (17,5%), *Eimeria auburnensis* (14,7%), *Eimeria ellipsoidallis* (9,7%), *Eimeria zuernii* (7,22%), *Eimeria brasiliensis* (4,56%), *Eimeria bukidnonensis* (3,97%), *Eimeria illinoisensis* (2,91%), *Eimeria wyomingensis* (1,42%), *Eimeria alabamensis* (1,27%), *Eimeria cylindrica* (0,76%), *Eimeria pellita* (0,54%), *Eimeria ildefonsoi* (0,21%) e *Eimeria subspherica* (0,07%). Concluiu-se que os bovinos do Semiárido do Brasil foram parasitados por 14 espécies de *Eimeria*, e acredita-se que o manejo sanitário empregado, bem como o sistema de criação desses animais, seja o ponto crucial para os altos índices de infecção nesta região.

## Introduction

Cattle farming occupies a prominent place in the worldwide agricultural scenario, and Brazil is one of the largest milk producers and meat exporters (Brasil, 2021). In the northeastern region of Brazil, beef cattle products and byproducts are used in food and commerce, thus generating stability and development (Carneiro, 2019). In the state of Paraíba, which is within this region of Brazil, cattle raising is also a viable activity that provides one of the main sources of animal protein for human consumption (IBGE, 2020). However, there are some obstacles to herd productivity. Among these, parasitism by enteric protozoa is responsible for diarrhea, weight loss and decreased meat and milk production, and may even lead to animal mortality in severe cases (Daugschies & Najdrowski, 2005; Dubey, 2019; Lopez-Osorio et al., 2020).

The main protozoa of veterinary medical interest belong to the phylum Apicomplexa. These are characterized by obligate intracellular parasitism that causes disease and destroys the host cells (Dubey, 2019). The genus *Eimeria* belongs to the class Sporozoasida, family Eimeriidae, and is transmitted by fecal-oral contamination. It develops in the epithelial cells of the digestive tract, where it causes an enteritis called eimeriosis or coccidiosis (Florião et al., 2016; Martins et al., 2020).

This disease becomes important because of the losses resulting from mortality among young animals and because of the reduced performance of those that recover from the infection, due to their lower food consumption and consequently diminished weight development (Abebe et al., 2008). Adult animals are mostly asymptomatic hosts, but serve as sources of infection for young animals, which are more susceptible to infections and may present gastrointestinal disorders and growth retardation (Daugschies & Najdrowski, 2005; Hillesheim & Freitas, 2016).

Several species of *Eimeria* are known to parasitize cattle. Some of them, such as *Eimeria zuernii* and *Eimeria bovis*, are classified as more pathogenic (Bangoura & Daugschies, 2007). Animals parasitized by these species have clinical signs associated with bloody diarrhea, dehydration, anorexia and weight loss; depending on the severity of infection, these animals may die (Cardoso et al., 2017).

*Eimeria alabamensis* and *Eimeria auburnensis* have been reported in outbreaks of moderately pathogenic clinical coccidiosis (Hillesheim & Freitas, 2016). *Eimeria ellipsoidalis* has been described as an occasional cause of diarrhea (Mielke et al., 1993). *Eimeria brasiliensis*, *Eimeria bukidnonensis*, *Eimeria canadensis*, *Eimeria cylindrica* and *Eimeria ildefonsoi* have been characterized as presenting low pathogenicity, such that they are manifested subclinically (Lima, 2004; Daugschies & Najdrowski, 2005; Das et al., 2015; Florião et al., 2016; Hillesheim & Freitas, 2016).

Therefore, identification of the various species of *Eimeria* spp. becomes very important. This enables focused disease control and prevention measures and favors adequate administration of medicines and disinfection of animal facilities in conventional production systems (Daugschies & Najdrowski, 2005), especially under conditions of higher stocking rates (Lima, 2004).

Considering the scarcity of information and the economic losses caused by coccidiosis, the objective of this study was to describe the diversity of species of *Eimeria* infecting cattle herds in Northeastern Brazil.

## Material and Methods

### Experimental design

**Figure 1:**
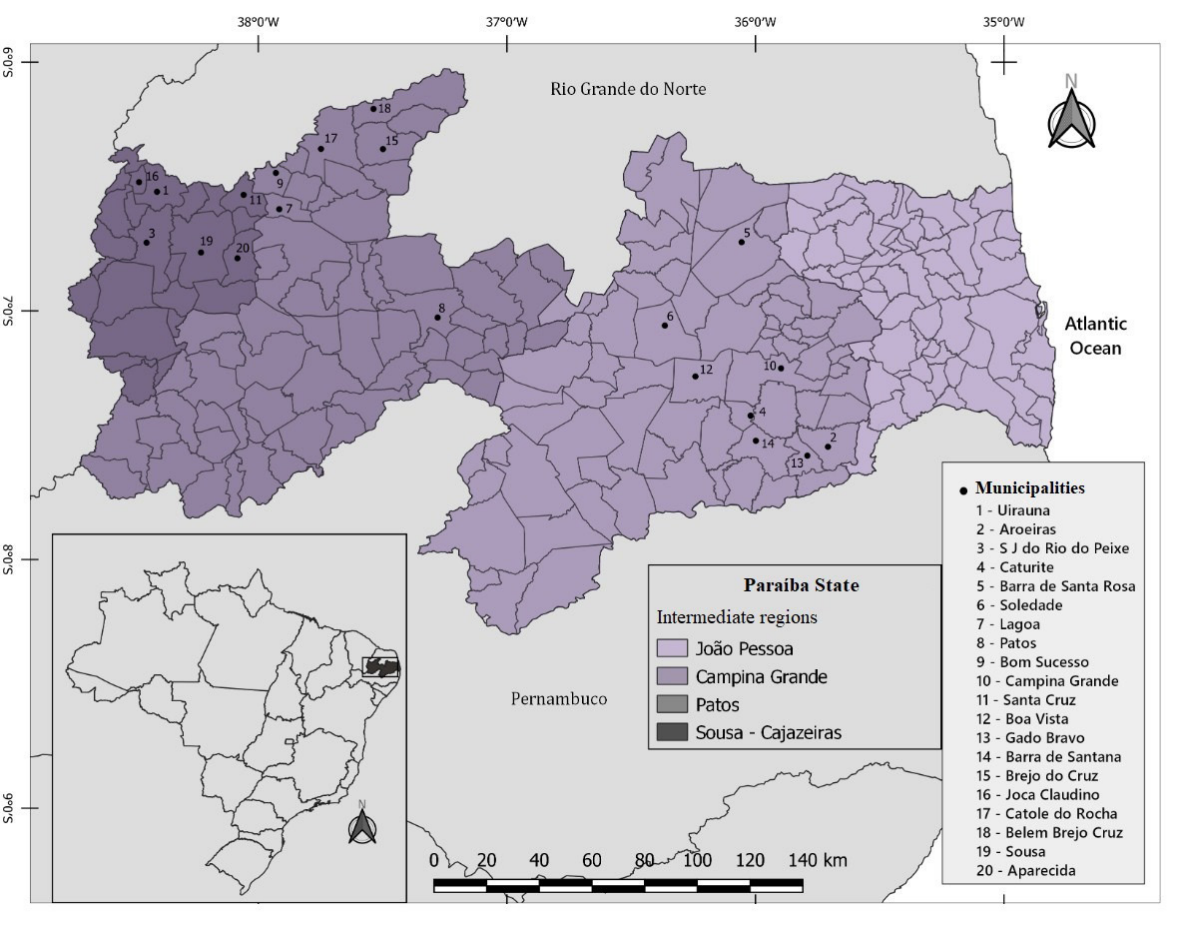
Geographical distribution of Municipalities in the Semiarid region of Paraíba, Northeastern Brazil, in which farms were visited to collect bovine feces.

Between January and December 2020, fecal sample collections were carried out on 20 farms that had cattle herds of more than 40 animals that were raised within a semi-intensive system. These farms were located at municipalities in the State of Paraíba that lie within the intermediate regions of Campina Grande, Patos and Sousa-Cajazeiras, which all have a semi-arid climate (Figure 1). The average annual precipitation in the State of Paraíba is between 250-800 mm. The rainfall is irregular and usually concentrated in the months of March to May. The maximum temperature is 32°C and the minimum is 20°C; the evaporation rates are high and the relative air humidity is close to 70%. The vegetation is predominantly that of the Caatinga biome (INMET, 2010).

Forty animals were randomly sampled from each of the 20 farms (which were all dairy farms), without distinction regarding their breed, sex or age, thus totaling 800 sampled animals.

### Sample collection and processing for analysis

Feces were collected directly from the rectum of the animals with the aid of clean plastic bags. The animals were identified according to their number, sex, age and farm. The samples were stored in isothermal boxes and were sent to the Veterinary Parasitology Laboratory (VPL) of the Veterinary Hospital of the Federal Institute of Paraíba (IFPB), Sousa, PB, Brazil, for further laboratory analysis.

To investigate the enteric protozoa, the centrifugation-floatation technique in sucrose solution was used, as originally described by Sheather (1923) and as modified by Duszynski & Wilber (1997). Through this, any presence of oocysts of *Eimeria* spp. was determined. Then, the feces of the positive animals were diluted in an aqueous solution of 2.5% potassium dichromate (K_2_Cr_2_O_7_), at a ratio of one-sixth feces to five-sixths solution. This mixture was placed in Petri dishes and left in a B.O.D. chamber at 28 ºC and relative humidity > 80% for 15 days, to await oocyst sporulation.

After this sporulation period, centrifugation-floatation was performed in a new sucrose solution. From this, a drop of the surface material was removed, placed on a slide under a coverslip, and the oocysts were viewed using a MAX-300 microscope with 40X and 100X objectives. This was coupled to a microcomputer through the MvImage® software, in the same way as described by Araújo et al. (2020).

From each positive sample, 20 intact sporulated oocysts of the genus *Eimeria* were photographed and measured in terms of the maximum, average and minimum diameters, and the Shape Index (SI) of these oocysts and sporocysts was calculated. In addition, the thickness of the oocyst wall was measured and the presence or absence of internal morphological structures was noted. To make morphological identifications of *Eimeria* species, the reference keys for sporulated oocysts described by Levine & Ivens (1967), Levine (1985), Duszynski & Wilber (1997), Berto et al. (2014) and Florião et al. (2016) were used.

### Statistical analysis

The mean diameter, lower limit, upper limit, standard deviation and coefficient of variation (CV) of the oocysts of *Eimeria* spp. and their sporocysts were evaluated using the Microsoft Office Excel 2010® software.

## Results

Oocysts of *Eimeria* spp. were found on 100% (20/20) of the farms visited. The presence of oocysts was detected in 17.12% (137/800) of the samples. It was found that 10.21% (14/137) of the cattle were parasitized by only one *Eimeria* species; 27% (37/137) were parasitized by two different species; 23.35% (32/137) had three species; 16.78% (23/137) were parasitized by four species; 14.59% (20/137) were parasitized by five species; 4.37% (6/137) were parasitized by six species; and 3.64% (5/137) were parasitized by seven different species of *Eimeria*.

**Figure 2:**
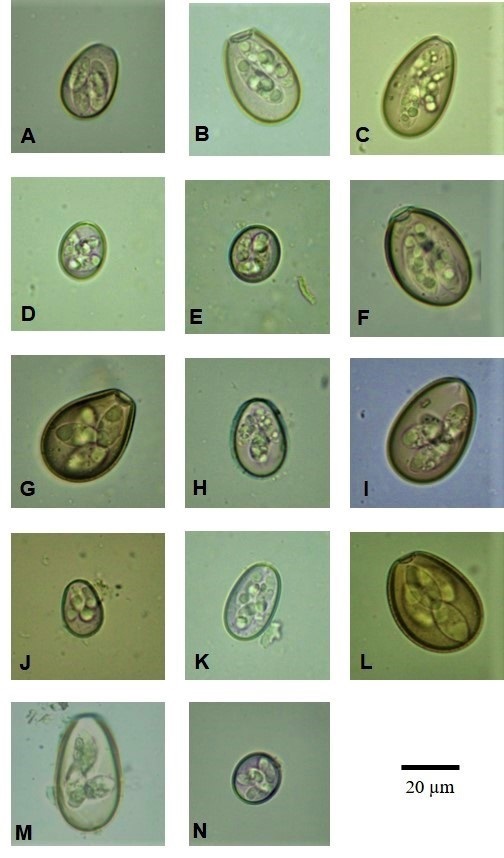
Photomicrographs of oocysts of *Eimeria* spp. in cattle in the Semiarid region of Paraíba, Northeastern Brazil. **A**: *Eimeria bovis;* **B:** *Eimeria canadensis*; **C:** *Eimeria auburnensis*; **D:** *Eimeria ellipsoidalis*; **E:** *Eimeria zuernii*; **F:** *Eimeria brasiliensis*; **G:** *Eimeria bukidnonensis*; **H:** *Eimeria illinoisensis*; **I:** *Eimeria wyomingensis*; **J:** *Eimeria alabamensis*; **K:** *Eimeria cylindrica*; **L:** *Eimeria pellita*; **M:** *Eimeria ildefonsoi*; **N:** *Eimeria subspherica.*

Based on the morphological characteristics of 2,740 intact sporulated oocysts, 14 species of the genus *Eimeria* were identified, which are listed here in descending order of frequency of occurrence: *E. bovis* Züblin, 1998 (Figure 2A); *E. canadensis* Bruce, 1921 (Figure 2B); *E. auburnensis* Cristenses and Poeter, 1939 (Figure 2C); *E. ellipsoidalis* Becker & Frye, 1929 (Figure 2D); *E. zuernii* Rivolta, 1878 (Figure 2E); *E. brasiliensis* Torres & Ramos, 1939 (Figure 2F); *E. bukidnonensis* Tubangui, 1939 (Figure 2G); *E. illinoisensis* Levine & Ivens, 1967 (Figure 2H); *E. wyomingensis* Huizinga & Winger, 1942 (Figure 2I); *E. alabamensis* Christenses, 1941 (Figure 2J); *E. cylindrica* Wilson, 1931 (Figure 2K); *E. pellita* Supperer, 1952 (Figure 2L); *E. ildefonsoi* Torres & Ramos, 1939 (Figure 2M); and *E. subspherica* Christenses, 1941 (Figure 2N).

The morphological characteristics and respective occurrence rates of the *Eimeria* species obtained in this study are described in Table 1. The percentages of occurrence and the mean values for the length, width, standard deviation, morphometric index, coefficient of variation and number of oocysts and sporocysts found in these *Eimeria* species are described in Table 2.

**Table 1:** Occurrence and morphology of oocysts and sporocysts of *Eimeria* spp. in cattle in the semiarid region of northeastern Brazil.

**Coccidia**	**Oocysts**				**Esporocysts**					
	**Shape**	**Polar granule**	**Outer layer**	**Micropyle**	**Shape**	**Polar granule**	**Stieda body**	**Residuum**	**Nº of cattle**	**Occurrence**
*E. bovis*	Ovoid	Present	Smooth	Present	Ovoid and cylindrical or elongated	Present	Present	Present	102	35.10%
*E. canadensis*	Ovoid or Ellipsoidal	Absent	Bi-layered	Present	Elongated and ellipsoidal	Absent	Present	Present	77	17.48%
*E. auburnensis*	Ovoid	Present	Bi-layered	Present	Elongated and ovoid	Present	Present	Present	71	14.70%
*E. ellipsoidalis*	Ellipsoidal	Present	Thin	Absent	Elongated and ellipsoidal	Present	Present	Present	51	9.70%
*E. zuernii*	Spherical	Absent	Single	Absent	Elongated and ovoid	Absent	Present	Present	45	7.22%
*E. brasiliensis*	Ellipsoidal or Ovoid	Optative	Bi-layered	Present	Elongated ellipsoid	Optative	Present	Present	16	4.56%
*E. bukidnonensis*	Piriform	Absent	Striated	Present	Elongated and ovoid	Absent	Present	Present	19	3.97%
*E. illinoisensis*	Ellipsoidal or Ovoid	Absent	Smooth	Absent	Ovoid elongated	Absent	Present	Present	18	2.91%
*E. wyomingensis*	Ovoid	Absent	Smooth	Present	Ellipsoid	Absent	Present	Absent	13	1.42%
*E. alabamensis*	Ovoid or Ellipsoidal	Optative	Smooth	Absent	Elongated and ovoid	Optative	Present	Absent	16	1.27%
*E. cylindrica*	Ellipsoidal elongated	Present	Thin	Absent	Elongated ellipsoid	Present	Present	Present	13	0.76%
*E. pellita*	Ovoid	Present	Thick	Present	Elongated	Present	Present	Present	4	0.54%
*E. ildefonsoi*	Ovoid or cylindrical	Absent	Bi-layered	Present	Ellipsoid	Absent	Present	Present	2	0.21%
*E. subspherica*	subspherical or Spherical	Optative	Bi-layered	Absent	Ovoid and spheric	Optative	Present	Present	2	0.07%

**Table 2:** Micrometric measurements of oocysts and sporocysts of *Eimeria* spp. infecting cattle in the semiarid region of Northeast Brazil.

**Coccidia**	**Oocysts**										**Sporocysts**						
	**Length (µm)**	**SD**	**CV (%)**	**Width (µm)**	**SD**	**CV (%)**	**SI**	**N**	**Length (µm)**	**SD**		**CV (%)**	**Width (µm)**	**SD**	**CV (%)**	**SI**	**N**
*E. bovis*	28 (24-32)	2.12	7.57	20 (17-23)	1.38	6.9	1.40	962	13 (8-19)	2.08		16	6-(4-8)	0.84	14	2.16	962
*E. canadensis*	35 (32-38)	1.55	4.43	22 (19-26)	1.09	4.95	1.59	479	16 (10-22)	2.12		13.5	7 (6-9)	0.89	12.71	2.28	479
*E. auburnensis*	37 (32-42)	2.41	6.51	22 (19-26)	1.34	6.09	1.68	403	16 (12-21)	2.15		13.44	7 (5-10)	0.90	12.86	2.28	403
*E. ellipsoidalis*	22 (19-26)	1.68	7.64	16 (13-19)	1.42	8.88	1.37	266	10 (5-16	1.93		19.3	6 (4-8)	0.83	13.83	1.66	266
*E. zuernii*	18 (15-22)	1.35	7.5	16 (13-19)	1.52	9.5	1.12	198	8 (4-13)	1.52		19	5 (4-7)	0.70	14	1.6	198
*E. brasiliensis*	35 (32-39)	1.56	4.46	26 (24-28)	1.07	4.12	1.34	125	16 (11-21)	2.57		16.06	7 (6-8)	0.70	10	2.28	125
*E. bukidnonensis*	42 (36-49)	3.45	8.21	30 (26-35)	2.84	9.47	1.40	109	14 (10-19)	2.06		14.71	9 (7-11)	0.95	10.56	1.55	109
*E. illinoisensis*	26 (23-29)	1.60	6.15	20 (18-22)	1.02	5.1	1.30	80	11 (6-17)	2.38		21.64	6 (5-7)	0.74	12.33	1.83	80
*E. wyomingensis*	41 (38-44)	1.53	3.73	27 (26-29)	0.64	2.37	1.51	39	16 (12-20)	3.05		19.06	7 (6- 9)	0.78	11.14	2.28	39
*E. alabamensis*	18 (14-23)	3.04	16.89	14 (12-16)	1.50	10.71	1.28	35	10 (7-13)	1.51		15.1	4 (3-6)	0.66	16.5	2.5	35
*E. cylindrica*	21 (20-23)	1.13	5.38	15 (13-17)	0.94	6.33	1.40	21	10 (8-12)	1.18		11.8	5 (4-7)	0.76	15.2	2	21
*E. pellita*	38 (36-41)	2.40	6.32	26 (25-28)	1.02	3.92	1.46	15	15 (13-19)	1.66		11.07	9 (8-11)	0.85	9.44	1.66	15
*E. ildefonsoi*	43 (43-44)	0.16	0.37	24 (24-25)	0.04	0.17	1.79	6	18 (18-19)	0.45		2.5	8 (8-9)	0.32	4	2.25	6
*E. subspherica*	15 (14-16)	1.69	11.27	14 (13-16)	2.54	18.14	1.07	2	6 (4-8)	3.39		56.5	4.2 (4-4.4)	0.35	8.33	1.42	2

## Discussion

The present study was the first to identify and describe distinct species of *Eimeria* parasitizing cattle in the semiarid region of Northeastern Brazil. Because of the high occurrence rates found, along with the presence of highly pathogenic species, we think that the number of cases of bovine coccidiosis was high. According to Hamid et al. (2019) and Dubey (2019), coccidiosis is distributed worldwide, reaching up to 100% of calves in the first weeks of age. Thus, it has a high impact on livestock development and economic results. The high incidence of infections by *Eimeria* spp. is associated with higher prevalence of the subclinical form, which therefore makes it difficult to assess the real economic impact caused by eimeriosis in cattle herds. The most frequent clinical sign is severe and/or hemorrhagic diarrhea; presence of the subclinical form is associated with less pathogenic species, low environmental pressure from more pathogenic species or an acquired immune response that already exists (Daugschies et al., 2007; Gillhuber et al., 2014).

This investigation also revealed that 14 species of *Eimeria* were present, which can be considered to be a high level of diversity. Lopez-Osorio et al. (2020) found similar diversity among cattle in different production systems in Colombia, in which identified 13 species of *Eimeria*. Seven species of *Eimeria* were also found by Das et al. (2015), in dairy cattle in India; and by Florião et al. (2016), on an organic dairy cattle farm in Rio de Janeiro, Brazil.

Although the occurrence rate of *Eimeria* spp. observed among the cattle studied here (17.12%; 137/832) can be considered high, it differed from the results found by Hillesheim & Freitas (2016), in the State of Paraná, Brazil, who reported that the prevalence of coccidia was 48.2% (53/110) among the animals evaluated, on family-run farms. The rate in the present study was also lower than the prevalences recorded by Lopez-Osorio et al. (2020) in Colombia and by Hastutiek et al. (2019) in Indonesia, with 75.5% (1006/1333) and 53.42% (190/357), respectively, in the cattle herds evaluated. We think that the semi-intensive rearing system used on the farms studied here may have had a relationship with the lower rates of infections found in the present study. Higher levels of coccidia are mainly related to fecal-oral contamination in feedlots (Kimeli et al., 2020). In addition, the high average annual temperatures and low rainfall to which oocysts are subjected in the environment of the semiarid climate may have led to reduction of their survival and consequent reduction of reinfection of the animals.

Notable numbers of species of *Eimeria* were identified parasitizing the same individuals. It can be highlighted that some animals (5/137) had up to seven different species. This demonstrates that there was high potential for coinfection among the cattle in the herds examined. Mixed infections were also observed by Abebe et al. (2008) in Ethiopia, ranging from two to eight species per animal. Amid the diversity of *Eimeria* species infecting the same animal, it is difficult to control these parasites through vaccines (Sultana et al., 2014; Dubey, 2019), since the variability of *Eimeria* spp. is very high. Studies have indicated that the real impact of coinfections by *Eimeria* spp. is still uncertain, considering that clinical signs of diarrhea in calves have been found only in association with single infections by *E. zuernii* or *E. bovis* (Bangoura et al., 2011; Enemark et al., 2013; Lopez-Osorio et al., 2020).

*Eimeria bovis* was the most frequently found species (35.1%), and it stood out as the most pathogenic species among the more than 20 species of *Eimeria* already described in cattle (Daugschies & Najdrowski, 2005; Bangoura & Daugschies, 2007; Deplazes et al., 2016). According to Hermosilla et al. (2012) and Lopez-Osorio et al. (2020), the clinical conditions caused by these species give rise to enteric infections that result in severe hemorrhagic typhlocolitis, clinically characterized by hemorrhagic and catarrhal diarrhea.

The other two most frequent species were *E. canadensis* (17.48%) and *E. auburnensis* (14.70%). This finding differed from the sequence observed by Vidal et al. (2013), who found that the species *E. ellipsoidalis* (39.7%), *E. alabamensis* (18.4%) and *E. bovis* (12.1%) predominated in calves in the state of Rio de Janeiro, in southeastern Brazil; and also differed from the findings of Hillesheim & Freitas (2016), in calves in Paraná, southern Brazil, where *E. bovis*, *E. auburnensis* and *E. alabamensis* were the most frequently encountered species, with rates of 23.6%, 11.8% and 9.1%, respectively. These species caused outbreaks of clinical coccidiosis of considerable pathogenicity.

*Eimeria canadensis*, *E. ellipsoidalis*, *E. brasiliensis*, *E. bukidnonensis*, *E. illinoisensis*, *E. wyomingensis*, *E. alabamensis*, *E. cylindrica*, *E. pellita*, *E. ildefonsoi* and *E. subspherica*, which were also described in the present study, are considered to be highly prevalent worldwide (Eckert et al., 1995; Florião et al., 2016). Even in the absence of clinical disease, cattle can be severely affected due to damage inflicted on intestinal tissue, thereby compromising the digestive process and general homeostasis, with adverse effects on wellbeing and animal performance (Daugschies & Najdrowski, 2005).

One important morphological tool that helps to differentiate the species of the genus *Eimeria* is the SI. This consists of dividing the largest diameter by the smallest. Oocysts may vary in size but their MIs show a rectilinear trend that reflects the volumetric shape of the sporulated oocysts. This SI is more precise for comparison between species than the average of dimensions, and also for comparing intraspecific variation (Long & Joyner, 1984; Vidal et al., 2013; Araújo et al., 2020).

Molecular tools are already being studied and are available for identifying oocysts of coccidia in cattle (Hastutiek et al., 2022). However, Lopez-Osorio et al. (2020) and Vidal et al. (2013) demonstrated that oocyst morphology is still an appropriate and reliable method for differentiating coccidia in epidemiological surveys. This was also shown by Araújo et al. (2020), through identifying species of coccidia in pigs in the semiarid region of northeastern Brazil.

Nonetheless, oocysts and/or sporocysts with MIs greater than 1.1 should always be described carefully. These may have a variety of shapes, such as ellipsoidal, ovoid or pear-shaped, *i.g.*: they form a so-called “ellipsoidal complex”. On the other hand, spherical oocysts usually have an MI of 1.0, while subspherical oocysts have MIs between 1.0 and 1.1 (Levine, 1985; Berto et al., 2014).

Our measurements of the length and width of the oocysts and sporocysts showed CVs below 25%. According to Siqueira & Tibúrcio (2011), CVs below 10% are considered low, while those above 30% are considered very high. In the current study, the CVs of oocyst length and width were lower than those of sporocysts, i.e. the oocysts were more homogeneous, thus only showing low to medium dispersion.

## Conclusion

The diversity of *Eimeria* species is high among cattle in the semiarid region of northeastern Brazil. *E. bovis*, which is considered to be the most pathogenic species, showed the highest frequency of occurrence. Multiple infections by up to seven species were found in the animals. The semi-intensive rearing system and the semiarid conditions to which oocysts are exposed may have been limiting factors regarding their survival. These conditions may therefore have acted towards reduction of reinfection among the animals, since, despite the high rate of occurrence of infections that was found, these values were lower than those reported from studies conducted in other locations.
